# Intraoperative Terlipressin During Liver Transplantation Is Associated with Reduced Vasoactive Requirements and Lower Postoperative Troponin Release

**DOI:** 10.3390/jcm15082916

**Published:** 2026-04-11

**Authors:** Przemysław Jasiewicz, Hubert Buchwald, Andrzej Kobryń, Marcin Schiller, Maciej Piankowski, Sonia Frieske, Stanisław Pierściński, Adam Arndt, Emilia Piotrowicz, Michał Wiciński, Maciej Słupski

**Affiliations:** 1 Department of Anaesthesiology and Intensive Therapy, Collegium Medicum, Nicolaus Copernicus University, 87-100 Torun, Poland; hbcmumk@proton.me (H.B.); marcin.schiller@gmail.com (M.S.); adamarndt89@gmail.com (A.A.); epiotrowicz19@gmail.com (E.P.); 2 Department of General, Hepatobiliary and Transplant Surgery, Collegium Medicum, Nicolaus Copernicus University, 87-100 Torun, Poland; drandrekob@yahoo.com (A.K.); mpiankowski@wp.pl (M.P.); soniafrieske@gmail.com (S.F.); stanpier@poczta.onet.pl (S.P.); maciejslupski@wp.pl (M.S.); 3 Department of Clinical Pharmacology, Collegium Medicum, Nicolaus Copernicus University, 87-100 Torun, Poland; wicinski4@wp.pl

**Keywords:** liver transplantation, terlipressin, vasoactive-inotropic score, hemodynamic instability, troponin, perioperative medicine

## Abstract

**Background/Objectives**: Intraoperative hemodynamic instability during liver transplantation (LT) is common and results from cirrhosis-related circulatory dysfunction and profound hemodynamic changes during graft reperfusion. High catecholamine requirements may contribute to secondary organ injury, including myocardial damage. Terlipressin, a selective vasopressin V1 receptor agonist, has been shown to improve hemodynamic stability during LT; however, the impact of a short, targeted intraoperative infusion on cardiac biomarkers remains unclear. **Methods**: This retrospective single-center study included adult patients undergoing elective orthotopic liver transplantation between May 2017 and December 2025. Emergency transplantations and retransplantations were excluded. All transplant procedures were performed by a single transplant surgeon, while anesthesia care was provided by multiple teams following standardized institutional protocols. Patients receiving a fixed intraoperative dose of terlipressin (0.85 mg administered over 10 min after portal vein clamping; *n* = 61) were compared with a control group not receiving terlipressin (*n* = 44). The primary outcome was the vasoactive-inotropic score (VIS), assessed intraoperatively and during the first three postoperative days. Secondary outcomes included postoperative high-sensitivity troponin I (Hs-TnI) concentrations measured on the day of surgery and on postoperative days 1 and 3. **Results**: Baseline demographic and clinical characteristics, including liver disease severity and baseline Hs-TnI, were comparable between groups. VIS values were significantly lower in the terlipressin group on the day of transplantation (14.3 ± 2.4 vs. 37.0 ± 5.0, *p* < 0.001) and on postoperative day 1 (10.4 ± 2.2 vs. 17.3 ± 3.4, *p* < 0.05). Differences were no longer significant on postoperative days 2 and 3. Postoperative Hs-TnI concentrations were significantly lower in the terlipressin group at all assessed time points, including day 0 (51.5 ± 11.3 vs. 150.4 ± 29.0 ng/L, *p* < 0.001), postoperative day 1 (124.7 ± 28.8 vs. 275.0 ± 74.0 ng/L, *p* < 0.05), and day 3 (51.1 ± 18.4 vs. 167.2 ± 54.2 ng/L, *p* < 0.05). **Conclusions**: In this retrospective cohort, intraoperative terlipressin administration was associated with lower perioperative vasoactive requirements and reduced postoperative troponin release. These findings suggest that targeted terlipressin administration during liver transplantation may contribute to improved perioperative hemodynamic stability. Prospective randomized studies are required to confirm these observations and determine their impact on clinically relevant outcomes.

## 1. Introduction

Intraoperative hemodynamic instability during liver transplantation arises from several pathophysiological mechanisms characteristic of both advanced liver cirrhosis and the surgical course of transplantation itself. First, patients with cirrhosis exhibit chronic hemodynamic alterations, including increased cardiac output, low systemic vascular resistance, and vasodilation leading to relative central hypovolemia [1,2]. Second, during the reperfusion phase, a sudden release of pro-inflammatory cytokines (e.g., TNF-α), potassium, acidotic metabolites, and cold preservation fluids from the graft into the recipient’s circulation can precipitate abrupt arterial hypotension, arrhythmias, or even cardiac arrest. Elevated cytokine concentrations correlate with increased catecholamine requirements to maintain adequate arterial pressure [3,4,5].

The consequences of intraoperative hemodynamic instability during liver transplantation primarily include an increased risk of acute kidney injury, primary graft dysfunction, perioperative mortality, and early postoperative multiorgan impairment [6,7]. Persistent hemodynamic instability may also lead to secondary organ injury (e.g., brain, heart), deterioration of graft function, prolonged hospitalization, and the need for intensive care [4,5,6,7].

To maintain adequate perfusion pressure and cardiac output, vasopressors (e.g., phenylephrine, terlipressin, norepinephrine) and inotropes (dopamine, dobutamine) are routinely used [4,8]. However, there are no universally accepted guidelines defining the optimal pharmacologic approach; drug selection depends largely on the patient’s hemodynamic profile and the specific intraoperative phase [8].

These agents, when used during liver transplantation, may contribute to cardiac injury, including acute heart failure and stress-induced cardiomyopathy, particularly in patients with latent cardiac dysfunction or following exposure to high catecholamine doses [1,9]. The underlying mechanisms involve abrupt hemodynamic shifts, elevated concentrations of endogenous and exogenous catecholamines, and cirrhosis-related predisposition. Events such as acute heart failure, arrhythmias, or Takotsubo-type cardiomyopathy have been reported most frequently during the reperfusion phase. Furthermore, catecholamine administration (norepinephrine, epinephrine) may impair hepatic graft perfusion and disrupt the microcirculation, thereby increasing the risk of organ injury, particularly in the liver and kidneys [10,11,12]. Cardiac troponin is a well-established biomarker of myocardial injury, widely used in the diagnosis of acute cardiovascular conditions, but it also correlates with subclinical myocardial dysfunction and cardiovascular risk, making it a valuable marker of perioperative myocardial injury [13].

Terlipressin, a long-acting vasopressin analogue with selective V1-receptor agonism, exerts a significant hemodynamic effect during liver transplantation by increasing mean arterial pressure (MAP) and systemic vascular resistance, stabilizing arterial pressure, and reducing the requirement for other vasopressors during critical surgical phases, especially graft reperfusion [14,15,16,17]. Its mechanism of action involves selective splanchnic vasoconstriction, resulting in decreased portal venous flow and pressure, thereby reducing the risk of intraoperative bleeding [15,16]. Terlipressin has been shown to reduce the incidence of severe post-reperfusion syndrome (PRS), stabilize hemodynamic parameters (MAP, HR), and allow for lower cumulative catecholamine use during vena cava clamping and reperfusion [14,16,17].

However, the clinical effect of a single short terlipressin infusion timed to the hemodynamically critical phase of portal vein clamping and early reperfusion has not yet been fully elucidated. We hypothesized that such a bolus could stabilize circulation, improve hemodynamic stability, and provide secondary cardioprotection, measurable through cardiac biomarkers including high-sensitivity troponin I (Hs-TnI).

The purpose of this paper was to evaluate the effect of intraoperative terlipressin administration on vasoactive requirements and postoperative troponin release in patients undergoing liver transplantation.

## 2. Materials and Methods

This retrospective single-center study was conducted at a tertiary academic liver transplant center between May 2017 and December 2025. Adult patients undergoing elective liver transplantation were included. The main indications for liver transplantation included end-stage liver disease due to cirrhosis of various etiologies, including viral hepatitis, alcohol-related liver disease, and other chronic liver conditions. Patients undergoing emergency transplantation or retransplantation were excluded from the analysis. During the study period, intraoperative terlipressin administration was implemented into the institutional anesthetic protocol, creating two cohorts of patients treated before and after protocol adoption.

All transplant procedures were performed by a single experienced transplant surgeon, ensuring surgical consistency, while anesthesia care was provided by multiple anesthesiology teams. All cases were conducted according to standardized institutional protocols followed by all anesthesia teams. All procedures were conducted under standardized general anesthesia with invasive monitoring and protocolized vasoactive management.

### 2.1. Anesthesia and Hemodynamic Monitoring

Anesthesia was induced with propofol (2 mg/kg), rocuronium (1.2 mg/kg), and an initial bolus of fentanyl (0.1 mg) to facilitate endotracheal intubation. Anesthesia was maintained with sevoflurane in an oxygen–air mixture, continuous rocuronium administration, and fentanyl infusion. After induction, bilateral radial arterial lines were placed for continuous invasive blood pressure monitoring and blood sampling. A central venous catheter was inserted into the right internal jugular vein. Standard anesthetic monitoring included electrocardiography, pulse oximetry, capnography, temperature monitoring, and urine output measurement. Hemodynamic parameters were continuously monitored using invasive arterial pressure, central venous pressure, and pulse contour cardiac output monitoring (FloTrac/Vigileo^®^, Edwards Lifesciences, Irvine, CA, USA), providing continuous assessment of cardiac output-derived variables.

### 2.2. Terlipressin Administration Protocol

During the study period, intraoperative terlipressin administration was introduced as part of the institutional anesthetic protocol for liver transplantation. Consequently, patients transplanted after implementation of this protocol routinely received terlipressin according to the predefined dosing strategy, whereas patients transplanted earlier in the study period did not receive terlipressin. The terlipressin protocol was developed based on available literature, including previously published studies on terlipressin use in liver transplantation [14,15]. In the terlipressin group (*n* = 61), a fixed total dose of 0.85 mg terlipressin (Glypressin^®^, Ferring Pharmaceuticals, Saint-Prex, Switzerland) was diluted in 50 mL of normal saline and administered via a central venous catheter over 10 min using a syringe pump. This dosing strategy was applied uniformly to all patients in the terlipressin group. The infusion was initiated immediately after portal vein clamping, corresponding to the phase of maximal splanchnic vasodilation during liver transplantation. No additional terlipressin doses were administered intraoperatively. The control group (*n* = 44) did not receive terlipressin or any other vasopressin analogue as part of the study protocol.

### 2.3. Vasoactive Management

In both groups, norepinephrine was used as the first-line vasopressor to maintain a mean arterial pressure (MAP) ≥ 65 mmHg. If additional hemodynamic support was required, epinephrine, dobutamine or milrinone could be administered at the discretion of the attending anesthesiologist. The use of vasopressin was recorded and incorporated into the vasoactive-inotropic score (VIS) calculation. All other aspects of intraoperative fluid management, transfusion thresholds, and anesthetic technique followed institutional standards. No patient received terlipressin outside of the predefined study protocol.

### 2.4. Hemodynamic and Biochemical Assessment

The vasoactive-inotropic score (VIS) was calculated at four predefined time points: during the intraoperative period (from induction of anesthesia to the end of surgery) and once daily during the first three postoperative days. For each time interval, the highest recorded VIS value was used for analysis to reflect peak hemodynamic support requirements. VIS was calculated using the following formula [18]:

VIS = dopamine (µg/kg/min) + dobutamine (µg/kg/min) + 100 × epinephrine (µg/kg/min) + 100 × norepinephrine (µg/kg/min) + 10 × milrinone (µg/kg/min) + 10,000 × vasopressin (U/kg/min)

Secondary outcomes included postoperative high-sensitivity troponin I (Hs-TnI), measured on the day of surgery and on postoperative days 1 and 3. All laboratory analyses were performed in the hospital’s central laboratory using standardized and validated assay methods.

### 2.5. Statistical Analysis

Statistical analyses were performed using Jamovi statistical software (version 2.6.44, The Jamovi Project). The distribution of continuous variables was assessed using the Shapiro–Wilk test, and most quantitative variables demonstrated non-normal distribution. Continuous variables are presented as mean ± standard error (SE) for descriptive clarity and comparability with previous studies in liver transplantation. However, due to the non-normal distribution of the data, between-group comparisons were performed using nonparametric tests, specifically the Mann–Whitney U test. Categorical variables were expressed as counts and compared using the chi-square test. For perioperative variables measured at predefined postoperative time points (VIS and Hs-TnI), comparisons between the terlipressin and control groups were performed independently for each time point using nonparametric testing due to the non-normal distribution of the data. Given the retrospective design, no formal a priori sample size calculation was performed, and all eligible patients during the study period were included in the analysis. A two-sided *p* value < 0.05 was considered statistically significant.

## 3. Results

### 3.1. Baseline Characteristics

Baseline demographic and clinical characteristics were comparable between the terlipressin and control groups. A total of 105 patients who underwent orthotopic liver transplantation were included in this retrospective analysis, of whom 61 received intraoperative terlipressin. There were no significant differences in age (52.4 ± 1.5 vs. 49.3 ± 1.7 years, *p* = 0.199) or sex distribution (*p* = 0.288). Height was similar between groups (174.8 ± 1.2 vs. 173.8 ± 1.3 cm, *p* = 0.515), while body weight was significantly higher in the terlipressin group (85.9 ± 2.2 vs. 79.2 ± 2.6 kg, *p* < 0.05), with a corresponding trend toward higher body mass index (28.0 ± 0.65 vs. 26.1 ± 0.7 kg/m^2^, *p* = 0.029). Baseline Hs-TnI concentrations did not differ between groups (8.7 ± 3.2 vs. 9.0 ± 4.1 ng/L, *p* = 0.744). Similarly, no significant differences were observed in baseline liver disease severity, as assessed by MELD score (11.2 ± 0.7 vs. 11.4 ± 0.8, *p* = 0.8), MELD-Na score (10.9 ± 0.8 vs. 12.5 ± 1.3, *p* = 0.367), or MELD 3.0 score (10.1 ± 0.8 vs. 9.8 ± 1.3, *p* = 0.592). Total operative time was also comparable between groups (376.6 ± 6.7 vs. 407.7 ± 23.8 min, *p* = 0.388) (Table 1).

### 3.2. Perioperative Hemodynamics

Vasoactive-Inotropic Score (VIS) differed significantly between groups in the early perioperative period. On the day of transplantation (Day 0), patients receiving intraoperative terlipressin exhibited markedly lower VIS values compared with the control group (14.3 ± 2.4 vs. 37.0 ± 5.0, *p* < 0.001). This difference persisted on postoperative day 1, with significantly lower VIS in the terlipressin group (10.4 ± 2.2 vs. 17.3 ± 3.4, *p* < 0.05).

On postoperative day 2, VIS remained numerically lower in the terlipressin group; however, the difference did not reach statistical significance (2.7 ± 0.8 vs. 9.2 ± 3.4, *p* = 0.148). By postoperative day 3, VIS values were low in both groups, and no significant difference was observed (0.6 ± 0.2 vs. 6.2 ± 3.3, *p* = 0.514) (Table 2).

### 3.3. Postoperative Cardiac Biomarkers

**Figure 1:**
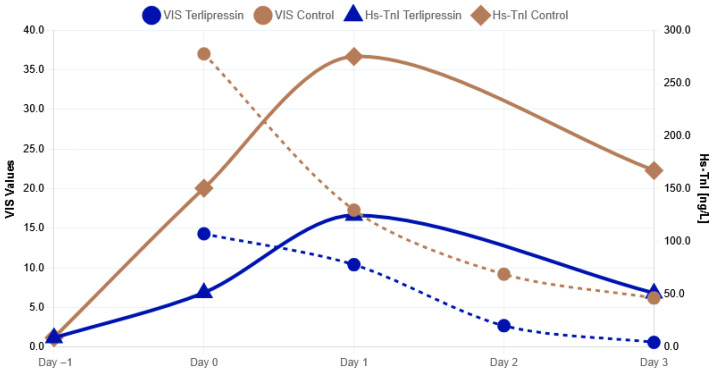
Perioperative changes in vasoactive-inotropic score (VIS) and high-sensitivity troponin I (Hs-TnI) in the terlipressin and control groups. Measurements were obtained before surgery (Day −1), intraoperatively (Day 0), and on postoperative days 1–3.

High-sensitivity troponin I (Hs-TnI) concentrations differed significantly between groups at all assessed perioperative time points. On the day of transplantation (Day 0), Hs-TnI levels were significantly lower in the terlipressin group compared with controls (51.5 ± 11.3 ng/L vs. 150.4 ± 29.0 ng/L, *p* < 0.001). This difference persisted on postoperative day 1, with lower Hs-TnI concentrations observed in patients receiving terlipressin (124.7 ± 28.8 ng/L vs. 275.0 ± 74.0 ng/L, *p* < 0.05). On postoperative day 3, Hs-TnI levels remained significantly lower in the terlipressin group (51.1 ± 18.4 ng/L vs. 167.2 ± 54.2 ng/L, *p* < 0.05) (Table 3 and Figure 1).

## 4. Discussion

This retrospective study found that a single short intraoperative terlipressin infusion during liver transplantation (LT) was associated with improved perioperative hemodynamic stability and lower postoperative cardiac biomarker release. Specifically, terlipressin use was linked to reduced vasoactive-inotropic requirements and attenuated elevations in high-sensitivity troponin I during the early postoperative period.

Previous studies have demonstrated that intraoperative terlipressin administration during LT primarily contributes to hemodynamic stabilization, a reduced incidence of severe post-reperfusion syndrome (PRS), and improved postoperative renal function. Randomized trials have shown that prophylactic terlipressin administration (e.g., 1 mg intravenously after portal vein clamping) significantly decreases the risk of severe PRS, reduces vasopressor requirements, and stabilizes arterial pressure, with additional benefits for early renal function. However, these effects may be accompanied by transient increases in pulmonary capillary wedge pressure and prolonged mechanical ventilation [14,15,16,19].

From a cardiovascular perspective, terlipressin increases systemic vascular resistance and may transiently raise afterload, potentially reducing cardiac output or ejection fraction, particularly in patients with advanced cirrhosis. These effects have raised concerns regarding possible subclinical myocardial injury [20]. Nevertheless, available data in the LT setting have not demonstrated an increased incidence of cardiac biomarker elevation or clinically significant ischemic cardiac events associated with terlipressin administration [14,15,19], findings that are consistent with the observations of the present study.

The present study has several strengths. First, it is based on a relatively homogeneous cohort of patients undergoing liver transplantation at a single center, ensuring consistency in perioperative management. Second, all procedures were conducted according to standardized institutional protocols, which reduced variability in anesthetic and perioperative care. Third, the study evaluates both intraoperative hemodynamic parameters and postoperative biochemical markers, providing a clinically relevant and integrated assessment of the effects of terlipressin administration.

### 4.1. Hemodynamic Effects

Lower VIS values reflect reduced catecholamine requirements and improved vascular tone, consistent with the V1 receptor–mediated vasoconstrictive effects of terlipressin. This hemodynamic profile may contribute to more stable arterial pressure during reperfusion, a period characterized by pronounced circulatory instability and recognized as a critical determinant of ischemia–reperfusion injury [21].

### 4.2. Cardiac Protection

Lower Hs-TnI concentrations observed in the terlipressin group suggest reduced perioperative myocardial stress, potentially related to improved hemodynamic stability and lower catecholamine exposure. Elevated cardiac troponin levels following LT have been associated with perioperative hypotension and catecholamine-mediated myocardial injury; stabilization of systemic vascular resistance with terlipressin may attenuate these contributing factors [22]. From a clinical perspective, lower postoperative cardiac biomarker release has been associated with improved postoperative cardiovascular outcomes [23]. These associations are of particular relevance in contemporary liver transplantation, given the increasing age and cardiovascular comorbidity burden of transplant recipients.

### 4.3. Clinical Perspective

Terlipressin is inexpensive, widely available, and does not require continuous titration. In the present study, a single fixed dose of 0.85 mg administered after portal vein clamping appeared feasible and reproducible within routine anesthetic practice. From a practical standpoint, terlipressin may represent a simple adjunct to perioperative hemodynamic management during liver transplantation, with minimal additional cost. Prospective studies are warranted to confirm these observations and to evaluate the impact of perioperative terlipressin administration on clinically relevant outcomes, including renal replacement therapy, duration of intensive care unit stay, and early allograft dysfunction.

### 4.4. Limitations

This study has several limitations inherent to its retrospective, single-center design, including the potential for selection bias and unmeasured confounding. Direct measures of tissue perfusion and microcirculatory flow were not available, limiting mechanistic interpretation of the observed hemodynamic effects. Long-term clinical outcomes were not assessed. The two study groups were separated chronologically due to the introduction of terlipressin into the institutional anesthetic protocol during the study period, and an era effect cannot be excluded. Furthermore, anesthesia care was provided by multiple anesthesiology teams, and although standardized protocols were applied, the potential influence of inter-operator variability cannot be fully excluded. Detailed data on postoperative complications, graft function parameters, and cardiovascular comorbidities were not consistently available and were therefore not included in the analysis. Assessment of myocardial injury was limited to serum troponin measurements, and future studies should incorporate additional diagnostic modalities, such as echocardiography, to provide a more comprehensive evaluation of cardiovascular function. Nevertheless, the uniform surgical technique performed by a single experienced transplant surgeon and the consistent timing and dosing of terlipressin administration strengthen internal consistency. The clear and reproducible biomarker trends observed across perioperative time points support the robustness of the associations identified.

## 5. Conclusions

In this retrospective analysis, a single intraoperative terlipressin infusion was associated with reduced vasoactive-inotropic requirements and attenuated postoperative cardiac biomarker release following liver transplantation. These findings suggest that perioperative terlipressin administration may offer a simple and low-cost adjunct to hemodynamic management during LT, with potential implications for end-organ protection. Prospective, randomized studies are required to confirm these observations, define the role of terlipressin within standardized perioperative transplantation protocols, and evaluate its potential impact on graft function outcomes.

## Figures and Tables

**Table 1:** Baseline Characteristics (Mean ± SE).

Marker	Terlipressin (*n* = 61)	Control (*n* = 44)	*p*
Age (years)	52.4 ± 1.5	49.3 ± 1.7	0.199
Sex	M: 46 F: 15	M: 29 F: 15	0.288
Weight (kg)	85.9 ± 2.2	79.3 ± 2.6	<0.05
Height (cm)	174.8 ± 1.2	173.8 ± 1.3	0.515
BMI (kg/m^2^)	28.0 ± 0.6	26.1 ± 0.7	<0.05
Hs-TnI (ng/L)	8.7 ± 3.2	9.0 ± 4.1	0.744
MELD	11.2 ± 0.7	11.4 ± 0.8	0.800
MELD-Na	10.9 ± 0.8	12.5 ± 1.3	0.367
MELD 3.0	10.1 ± 0.8	9.8 ± 1.3	0.592
Time (min)	376.6 ± 6.7	407.7 ± 23.8	0.388

**Table 2:** Vasoactive-Inotropic Score (VIS) During Surgery and in the Early Postoperative Period in the Terlipressin and Control Groups.

VIS	Terlipressin (*n* = 61)	Control (*n* = 44)	*p*
Day 0	14.3 ± 2.4	37.0 ± 5.0	<0.001
Postoperative 1	10.4 ± 2.2	17.3 ± 3.4	<0.05
Postoperative 2	2.7 ± 0.8	9.2 ± 3.4	0.148
Postoperative 3	0.6 ± 0.2	6.2 ± 3.3	0.514

**Table 3:** High-Sensitivity Troponin I (Hs-TnI) During Surgery and in the Early Postoperative Period in the Terlipressin and Control Groups.

Hs-TnI	Terlipressin (*n* = 61)	Control (*n* = 44)	*p*
Day 0	51.5 ± 11.3	150.4 ± 29.0	<0.001
Postoperative 1	124.7 ± 28.8	275.0 ± 74.0	<0.05
Postoperative 3	51.1 ± 18.4	167.2 ± 54.2	<0.05

## Data Availability

The data presented in this study are available on request from the corresponding author. The data are not publicly available due to institutional and patient privacy restrictions.

## Abbreviations

The following abbreviations are used in this manuscript:

LT	Liver transplantation
VIS	Vasoactive-inotropic score
Hs-TnI	High-sensitivity troponin I
MAP	Mean arterial pressure
HR	Heart rate
PRS	post-reperfusion syndrome
SE	Standard error
MELD	Model of End-Stage Liver Disease
